# Development and validation of a machine learning model based on multi-source clinical data for predicting the risk of early neurological deterioration in patients with ischemic stroke

**DOI:** 10.3389/fneur.2026.1911108

**Published:** 2026-09-15

**Authors:** Yue Li, Wei Wang, Yilan Wei, Jing Han, Yuan Shi, Quping Ouyang

**Affiliations:** Neurological Disease Center, Beijing Shunyi District Hospital, Beijing, China

**Keywords:** early neurological deterioration, logistic regression, machine learning, prediction model, stroke in progression

## Abstract

**Background and objective:**

Early neurological deterioration (END) is a critical clinical event associated with poor patient outcomes after acute ischemic stroke. Early identification of high-risk patients is crucial for timely clinical management. This study aimed to develop and validate a model for predicting END risk for acute ischemic stroke patients using machine learning algorithms.

**Methods:**

This study retrospectively and consecutively enrolled 1,151 patients with acute ischemic stroke from the Stroke Center of Beijing Shunyi District Hospital between January 2021 and December 2024. END was defined as progressive worsening of neurological deficit symptoms after onset. Predictive variables were screened using univariate analysis and multiple feature selection methods (Treebag, Boruta, Bayesian). Nine machine learning algorithms (Decision Tree, Efficient Neural Network, K-Nearest Neighbors, Light Gradient Boosting Machine, Logistic Regression, Multilayer Perceptron, Random Forest, Simplified Support Vector Machine, Extreme Gradient Boosting) were employed to construct prediction models. Hyperparameters were optimized via 10-fold cross-validation, and model performance was evaluated in an internal validation cohort (30% of the sample). Primary evaluation metrics included the area under the receiver operating characteristic curve (AUC), sensitivity, specificity, accuracy, F1 score, and net benefit from decision curve analysis (DCA). The SHAP method was used to interpret the optimal model.

**Results:**

A total of 161 patients (14.0%) developed END. Feature selection ultimately identified five key predictors: ischemic stroke etiological subtype, Oxford Community Stroke Project (OCSP) classification, age, atrial fibrillation history, and prior stroke history. In both the development and internal validation cohorts, the logistic regression model demonstrated favorable and stable performance (development cohort AUC: 0.787, 95% CI: 0.735–0.839; internal validation cohort AUC: 0.751, 95% CI: 0.668–0.834), with a low log-loss value. DCA suggested potential clinical utility of the logistic regression model. SHAP analysis revealed that the etiological subtype of ischemic stroke and age were the features contributing most to the model’s predictions.

**Conclusion:**

This study successfully developed and validated a logistic regression model for predicting END risk. The model incorporates five routinely available clinical variables and demonstrates satisfactory predictive performance and interpretability. The developed online tool may assist clinicians in early risk stratification, providing a reference for personalized intervention.

## 1 Introduction

Stroke is one of the leading global causes of disability and mortality (1–3). Early neurological deterioration (END) refers to a decline in neurological function occurring within hours or days after the onset of acute ischemic stroke (4, 5). END may manifest as worsening of pre-existing deficits, including increased limb weakness, worsening language or speech impairment, or a reduced level of consciousness, or as the development of new focal neurological deficits (6, 7). END is closely associated with poorer clinical outcomes, higher mortality, and a greater burden of long-term disability. Therefore, accurately identifying patients at high risk for END during the early phase of acute ischemic stroke is of significant clinical importance for the timely adjustment of treatment strategies, optimization of medical resource allocation, and individualized clinical management. Machine Learning (ML) can integrate multidimensional clinical data and identify complex associations related to the risk of END. Previous studies have demonstrated its potential for END prediction. For example, Sung et al. integrated clinical and neuroimaging information and compared several algorithms, including Boosted Trees, deep neural networks, and logistic regression, to predict the risk of END in patients with acute minor stroke (8). Subsequently, a two-center study of older patients with stroke combined lesion-location and radiomic features, and its random forest model maintained favorable predictive performance in an external validation cohort (9). Another study further integrated clinical, laboratory, and imaging variables to develop a random forest-based model for predicting END (10). However, many existing models are population-specific or rely on complex imaging features, limiting their broader applicability and clinical implementation. Moreover, model parsimony, interpretability, and clinical deployability have not been consistently combined. Therefore, a clinically practical END prediction model based on routinely available variables is still needed. To address these practical gaps, we used routinely available multidimensional clinical data, including demographics, medical history, clinical assessments, and treatment information, to develop and internally validate END prediction models. Multiple feature-selection methods and ML algorithms were compared, and the model with the best overall performance was selected. Model interpretation techniques were then applied to clarify its decision-making basis. Ultimately, an online prediction tool support early individualized END risk assessment in patients with acute ischemic stroke.

## 2 Methods

### 2.1 Study design and participants

This was a single-center, retrospective cohort study based on the stroke-center registry of Beijing Shunyi District Hospital. We reviewed the electronic medical records of all patients admitted with acute ischemic stroke between January 2021 and December 2024. Patients were eligible if they were aged 18 years or older; had a first or recurrent ischemic stroke with a pre-stroke modified Rankin Scale (mRS) score of 0–2; were admitted within 72 h of symptom onset; and had complete baseline data and serial neurological assessments. We excluded patients with transient ischemic attack (TIA); neurological deficits attributable to intracranial tumor, trauma, or arteriovenous malformation; those who died or left against medical advice within 24 h of admission; and those with more than 20% of key variables missing. After applying these criteria, 1,151 of 3,512 screened patients were included (Figure 1). The study was approved by the Ethics Committee of Beijing Shunyi District Hospital (approval no. 2023-K-024), which waived individual informed consent given the retrospective design and the use of de-identified data.

**Figure 1:**
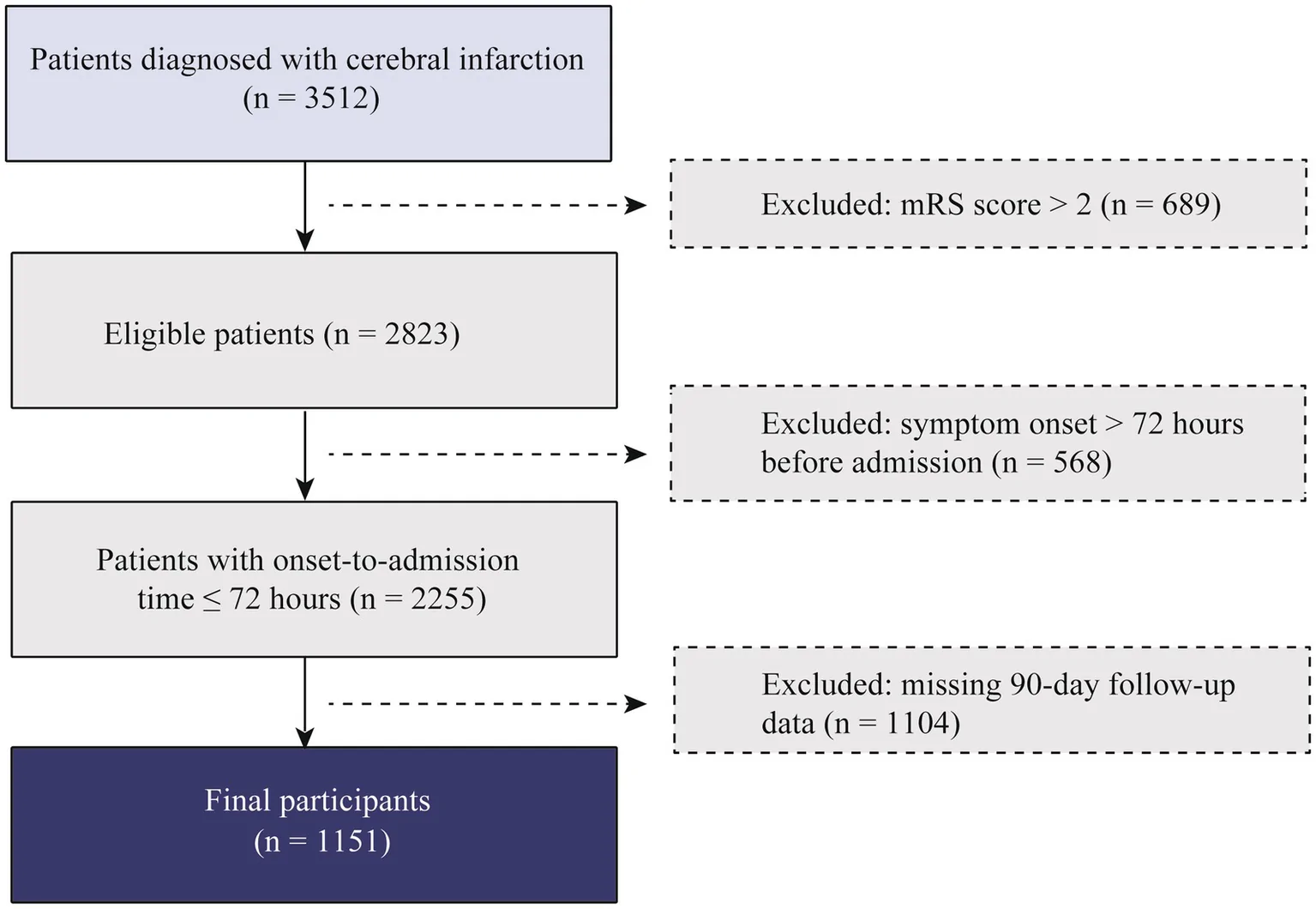
The flowchart of baseline analysis participant selection. Flow diagram of participant selection for the baseline analysis. Among 3,512 patients diagnosed with cerebral infarction, 689 with a modified Rankin Scale (mRS) score >2 were excluded, leaving 2,823 eligible patients. After excluding 568 patients with an onset-to-admission time >72 h and 1,104 patients with missing 90-day follow-up data, 1,151 patients constituted the final analytic cohort. mRS, modified Rankin Scale.

### 2.2 Outcome definition

The primary outcome was END. END was defined as an increase of at least 4 points in the total National Institutes of Health Stroke Scale (NIHSS) score relative to the admission baseline, a clinically meaningful worsening of individual NIHSS components (for example, level of consciousness or limb motor power), or the appearance of new focal neurological deficits, occurring within the first 7 days after admission (11, 12). Events with an identifiable secondary cause—hemorrhagic transformation, new or recurrent infarction, or a systemic or metabolic disturbance—were excluded by repeat brain CT or MRI together with clinical review. Outcome status was adjudicated by attending neurologists using the serial NIHSS assessments recorded in the registry.

### 2.3 Predictor variables

Candidate predictors were selected for their clinical relevance and ready availability at the bedside, and were grouped into three domains: baseline demographic and vascular factors (age, sex, history of atrial fibrillation, TIA, hypertension, diabetes, hyperlipidemia, recent smoking, history of prior stroke, and systolic and diastolic blood pressure before thrombolysis); admission examination and laboratory findings (pre-stroke mRS, blood glucose and white-cell count before thrombolysis, ischemic-stroke etiological subtype, and Oxfordshire Community Stroke Project [OCSP] classification); and medication exposure (aspirin, clopidogrel, and oral antihypertensive agents). All variables were objective, registry-recorded items. “History of confirmed stroke” referred to a previously documented physician diagnosis of stroke and was distinct from the index event.

### 2.4 Data preprocessing

Continuous variables were screened for outliers with boxplots (Supplementary Figure S1). Values lying more than 1.5 interquartile ranges above the third quartile or below the first quartile were treated as outliers and were winsorized to the corresponding fence; this bounded extreme but potentially valid physiological readings rather than discarding them. Missing values were few overall (≤5% for each variable; Supplementary Figure S2) and were handled by multiple imputation with chained equations (mice package in R). All candidate predictors and the outcome (END) were included as auxiliary variables in the imputation model, whereas the outcome itself was never imputed. The imputed and observed distributions agreed closely (Supplementary Table S1). Laboratory measurements were retained as continuous variables, and categorical predictors were specified before modeling. To obtain an unbiased estimate of generalization, the analytic dataset was split at random into a development set (70%, *n* = 806) and an independent hold-out validation set (30%, *n* = 345), stratified by outcome.

### 2.5 Feature selection

To limit the selection bias inherent in any single algorithm, features were screened with three complementary methods—Treebag (bagged-tree importance) (13), Boruta (comparison against permuted “shadow” features) (14), and a Bayesian feature-selection procedure (15)—and the variables retained by all three were intersected through a Venn approach. This ensemble set was then intersected with the variables that reached significance in univariate logistic regression, giving the final predictor set. The stability of selection was assessed with 10 rounds of 10-fold cross-validation on the development set.

### 2.6 Model development

Nine machine-learning algorithms were trained to predict END: decision tree (DT), elastic net (ENet), k-nearest neighbors (KNN), light gradient boosting machine (LightGBM), logistic regression, multilayer perceptron (MLP), random forest (RF), reduced support vector machine (RSVM), and extreme gradient boosting (XGBoost). For each algorithm, a model-specific hyperparameter grid was tuned on the development set by minimizing log-loss over 10 rounds of 10-fold cross-validation restricted to the final feature subset; the optimized hyperparameters are listed in Supplementary Table S2. Because END was relatively uncommon (14% of the cohort), model comparison relied on threshold-independent and imbalance-aware metrics rather than on raw accuracy.

### 2.7 Model evaluation

Each model was evaluated on both the development set and the independent hold-out validation set. Discrimination was summarized by the area under the receiver operating characteristic curve (AUC), with bias-corrected 95% confidence intervals (CIs) obtained from 1,000 bootstrap resamples, and AUCs were compared between models with DeLong’s test (16, 17). Calibration was assessed with calibration curves and log-loss. Classification performance was reported as sensitivity, specificity, positive and negative predictive values, accuracy, balanced accuracy, F1 score, Matthews correlation coefficient, and Youden’s index. Clinical usefulness was examined with decision curve analysis (DCA) (18), and the incremental value of one model over another was quantified with the net reclassification improvement (NRI) and integrated discrimination improvement (IDI) (19). Two-sided *p* < 0.05 was taken as significant.

### 2.8 Model interpretation

The final model was interpreted with SHapley Additive exPlanations (SHAP) (20), which attributes each patient’s prediction to its input features. Global (feature-level) and local (patient-level) explanations were derived from mean absolute SHAP values, summary and dependence plots, and individual waterfall plots.

### 2.9 Web calculator

The final model was embedded in a browser-based calculator (R Shiny) that returns the predicted probability of END from the five input variables.

### 2.10 Statistical analysis

All analyses were performed in R 4.4.2. Continuous variables are presented as mean (SD) and compared with the t test, or as median (interquartile range) and compared with the Wilcoxon rank-sum test; categorical variables are presented as count (%) and compared with the *χ*^2^ or Fisher’s exact test. Univariate and multivariable logistic regression were used to identify factors associated with END in the full cohort, and the dose–response relationship between age and END was examined with restricted cubic splines (RCS), with knots placed at recommended percentiles and the overall association and nonlinearity tested by Wald tests. The 70:30 partition was an internal random split and is referred to throughout as an independent hold-out validation set rather than as external validation.

## 3 Results

### 3.1 Baseline characteristics

The final cohort comprised 1,151 patients with acute ischemic stroke, of whom 161 (14.0%) developed END within 7 days. The development and validation sets were comparable in case mix: the proportion of END was the same in both (113/806, 14.0%; 48/345, 13.9%; *p* = 0.96), and most baseline variables did not differ between them (Table 1).

**Table 1:** Baseline characteristics of early neurological deterioration between training and validation sets.

Variable	Overall	Early neurological deterioration		*p*-value^2^	Datasets		*p*-value^2^
		Non early neurological deterioration	Early neurological deterioration		Training set	Validation set	
	*N* = 1,151^1^	*N* = 990^1^	*N* = 161^1^		*N* = 806^1^	*N* = 345^1^	
Group							0.96
Non early neurological deterioration	990 (86%)				693 (86%)	297 (86%)	
Early neurological deterioration	161 (14%)				113 (14%)	48 (14%)	
Age	64 (12)	64 (12)	68 (12)	<0.001	64 (12)	65 (11)	0.1
Gender				0.66			0.43
Male	775 (67%)	669 (68%)	106 (66%)		537 (67%)	238 (69%)	
Female	376 (33%)	321 (32%)	55 (34%)		269 (33%)	107 (31%)	
History atrial fibrillation				<0.001			0.94
No	1,052 (91%)	924 (93%)	128 (80%)		737 (91%)	315 (91%)	
Yes	99 (8.6%)	66 (6.7%)	33 (20%)		69 (8.6%)	30 (8.7%)	
History TIA				0.44			0.07
No	1,102 (96%)	946 (96%)	156 (97%)		766 (95%)	336 (97%)	
Yes	49 (4.3%)	44 (4.4%)	5 (3.1%)		40 (5.0%)	9 (2.6%)	
History hypertension				0.48			0.32
No	378 (33%)	329 (33%)	49 (30%)		272 (34%)	106 (31%)	
Yes	773 (67%)	661 (67%)	112 (70%)		534 (66%)	239 (69%)	
History diabetes				0.26			0.41
No	856 (74%)	742 (75%)	114 (71%)		605 (75%)	251 (73%)	
Yes	295 (26%)	248 (25%)	47 (29%)		201 (25%)	94 (27%)	
History hyperlipidemia				0.82			0.47
No	788 (68%)	679 (69%)	109 (68%)		557 (69%)	231 (67%)	
Yes	363 (32%)	311 (31%)	52 (32%)		249 (31%)	114 (33%)	
Smoked				0.49			0.75
No	679 (59%)	580 (59%)	99 (61%)		473 (59%)	206 (60%)	
Yes	472 (41%)	410 (41%)	62 (39%)		333 (41%)	139 (40%)	
Stroke				0.006			0.14
No	754 (66%)	664 (67%)	90 (56%)		539 (67%)	215 (62%)	
Yes	397 (34%)	326 (33%)	71 (44%)		267 (33%)	130 (38%)	
SBP before thrombolysis, mmHg	352 (1,755)	369 (1,836)	242 (1,130)	0.19	404 (1,986)	229 (1,023)	0.14
DBP before thrombolysis, mmHg	86 (14)	86 (13)	87 (15)	0.34	86 (14)	86 (13)	0.72
Before mRS				<0.001			0.45
Asymptomatic	829 (72%)	728 (74%)	101 (63%)		586 (73%)	243 (70%)	
No significant	188 (16%)	162 (16%)	26 (16%)		133 (17%)	55 (16%)	
Mild	109 (9.5%)	85 (8.6%)	24 (15%)		68 (8.4%)	41 (12%)	
Moderate	18 (1.6%)	11 (1.1%)	7 (4.3%)		14 (1.7%)	4 (1.2%)	
Moderate severe	2 (0.2%)	0 (0%)	2 (1.2%)		1 (0.1%)	1 (0.3%)	
Severe	5 (0.4%)	4 (0.4%)	1 (0.6%)		4 (0.5%)	1 (0.3%)	
Glucose before thrombolysis, mmol/L	14.6 (203.5)	15.5 (219.4)	9.3 (4.1)	0.002	17.1 (243.2)	8.7 (4.4)	0.61
WB before thrombolysis, 1,000 cells/uL	8.08 (10.28)	8.10 (11.02)	7.96 (3.09)	0.23	8.19 (12.09)	7.81 (3.33)	0.69
Subtypes for IS				<0.001			0.66
Arterial atherosclerosis	542 (47%)	421 (43%)	121 (75%)		376 (47%)	166 (48%)	
Lacunar infarction	332 (29%)	319 (32%)	13 (8.1%)		241 (30%)	91 (26%)	
Cardioembolism	45 (3.9%)	26 (2.6%)	19 (12%)		31 (3.8%)	14 (4.1%)	
Others	232 (20%)	224 (23%)	8 (5.0%)		158 (20%)	74 (21%)	
OCSP type				<0.001			0.99
Partial anterior circulation	452 (39%)	355 (36%)	97 (60%)		318 (39%)	134 (39%)	
Lacunar infarction	327 (28%)	316 (32%)	11 (6.8%)		227 (28%)	100 (29%)	
Posterior circulation infarction	144 (13%)	120 (12%)	24 (15%)		99 (12%)	45 (13%)	
Total anterior circulation	36 (3.1%)	15 (1.5%)	21 (13%)		26 (3.2%)	10 (2.9%)	
Others	192 (17%)	184 (19%)	8 (5.0%)		136 (17%)	56 (16%)	
Aspirin				0.19			0.33
No	950 (83%)	823 (83%)	127 (79%)		671 (83%)	279 (81%)	
Yes	201 (17%)	167 (17%)	34 (21%)		135 (17%)	66 (19%)	
Clopidogrel				0.38			0.97
No	1,114 (97%)	960 (97%)	154 (96%)		780 (97%)	334 (97%)	
Yes	37 (3.2%)	30 (3.0%)	7 (4.3%)		26 (3.2%)	11 (3.2%)	
Antihypertensive drugs				0.96			0.38
No	570 (50%)	490 (49%)	80 (50%)		406 (50%)	164 (48%)	
Yes	581 (50%)	500 (51%)	81 (50%)		400 (50%)	181 (52%)	

^1^Mean (sd) or Frequency (%).

^2^Wilcoxon rank sum test; Pearson’s Chi-squared test; Fisher’s exact test.

Several features distinguished patients who developed END from those who did not (Table 1). Patients with END were older (mean [SD], 68 [12] vs. 64 [12] years; *p* < 0.001) and more often had a history of atrial fibrillation (33 [20%] vs. 66 [6.7%]; *p* < 0.001) and of previously confirmed stroke (71 [44%] vs. 326 [33%]; *p* = 0.006). Pre-stroke disability was greater in the END group, which contained proportionally fewer asymptomatic patients (101 [63%] vs. 728 [74%]) and more patients with moderate-to-severe disability. By etiological subtype, large-artery atherosclerosis (121 [75%] vs. 421 [43%]) and cardioembolism (19 [12%] vs. 26 [2.6%]) were over-represented among END patients, whereas lacunar infarcts were under-represented (13 [8.1%] vs. 319 [32%]; overall *p* < 0.001). The OCSP distribution followed the same pattern: END patients more often had partial anterior circulation infarcts (97 [60%] vs. 355 [36%]) and total anterior circulation infarcts (21 [13%] vs. 15 [1.5%]), and less often had lacunar syndromes (11 [6.8%] vs. 316 [32%]; overall *p* < 0.001). Sex, admission blood pressure, and the vascular comorbidities other than atrial fibrillation did not differ between the two groups.

### 3.2 Factors associated with END

In univariate logistic regression on the full cohort, five variables were associated with END (Supplementary Table S3): age (odds ratio [OR] 1.03 per year, 95% CI 1.01–1.04; *p* < 0.001), atrial fibrillation (OR 0.28 for absence vs. presence, 0.18–0.44; *p* < 0.001), previously confirmed stroke (OR 0.62, 0.44–0.87; *p* = 0.006), ischemic-stroke etiological subtype (for example, cardioembolism OR 2.54, 1.36–4.75; lacunar infarct OR 0.14, 0.08–0.26; both vs. large-artery atherosclerosis; *p* < 0.001), and OCSP classification (total anterior circulation infarct OR 5.12, 2.55–10.31; lacunar syndrome OR 0.13, 0.07–0.24; both vs. partial anterior circulation infarct; *p* < 0.001). Admission glucose was not associated with END (*p* = 0.77).

### 3.3 Feature selection

The three selection methods each retained a largely overlapping set of variables, and their intersection comprised ischemic-stroke etiological subtype, admission glucose, OCSP classification, age, atrial-fibrillation history, and previously confirmed stroke (Supplementary Figure S3). Intersecting this set with the significant univariate predictors removed admission glucose—consistent with its null univariate association—and left five variables for modeling: ischemic-stroke etiological subtype, OCSP classification, age, atrial-fibrillation history, and previously confirmed stroke.

### 3.4 Age and END: dose–response

Restricted cubic spline analysis showed that the probability of END rose with age in an approximately linear fashion (*p* for overall association = 0.001), with no evidence of a nonlinear component (*p* for nonlinearity = 0.325; Figure 2). The fitted curve began to climb more steeply from around 73 years, although the formal test did not support a threshold effect.

**Figure 2:**
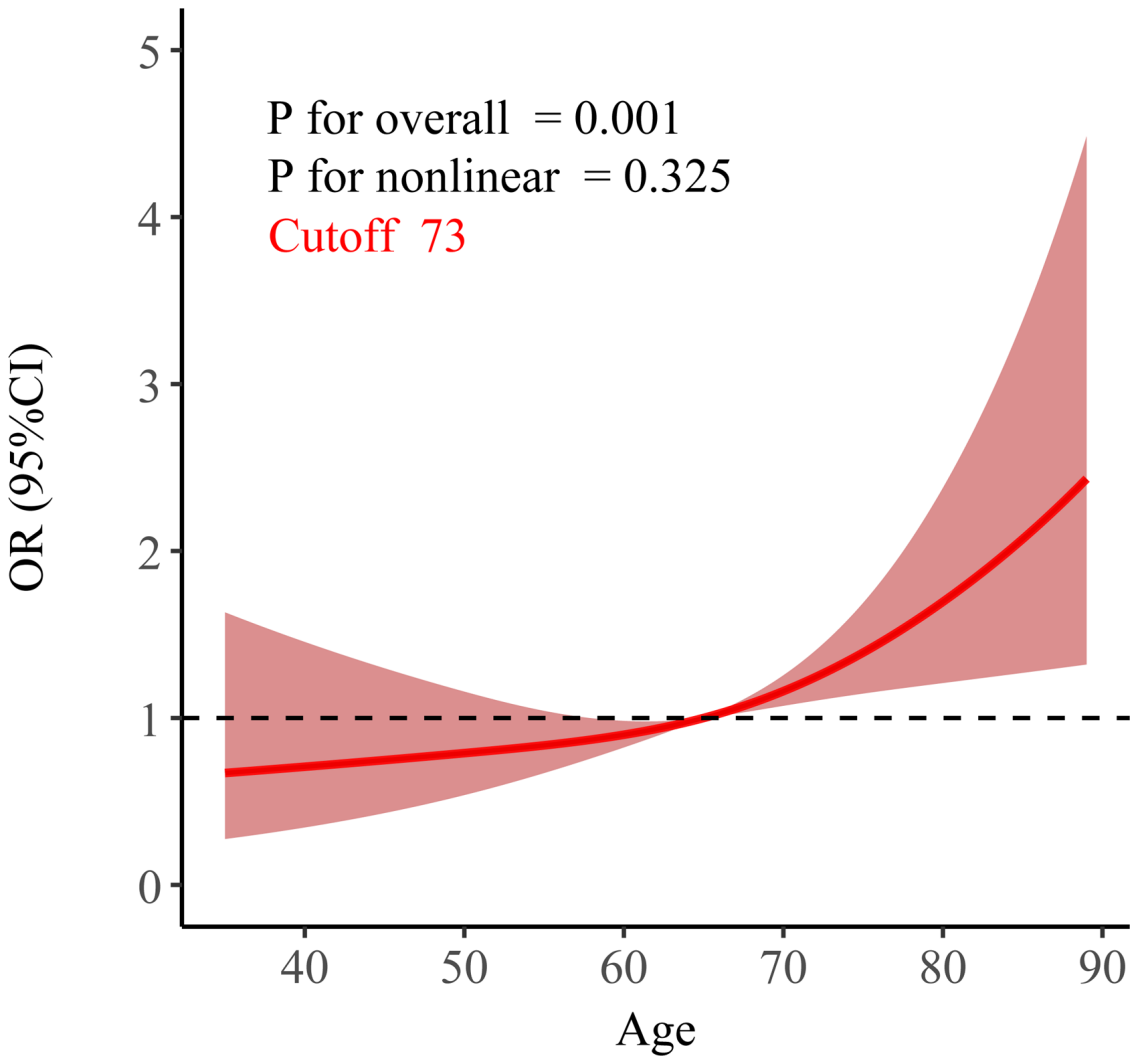
Restricted cubic spline (RCS) plots for different continuous variables. Restricted cubic spline (RCS) analysis of the relationship between age and the risk of early neurological deterioration (END). The solid curve shows the estimated odds ratio (OR) with its 95% confidence interval (shaded band); the dashed horizontal line indicates an OR of 1 (no association). The overall association was statistically significant (*p* = 0.001), whereas the non-linear component was not (*p* = 0.325). END, early neurological deterioration; OR, odds ratio; RCS, restricted cubic spline.

### 3.5 Model performance and selection

In 10 × 10-fold cross-validation on the development set, four models clustered at the top for discrimination: MLP (AUC 0.771, 95% CI 0.699–0.843), LightGBM (0.770, 0.709–0.831), ENet (0.767, 0.694–0.840), and logistic regression (0.764, 0.688–0.840; Figure 3).

**Figure 3:**
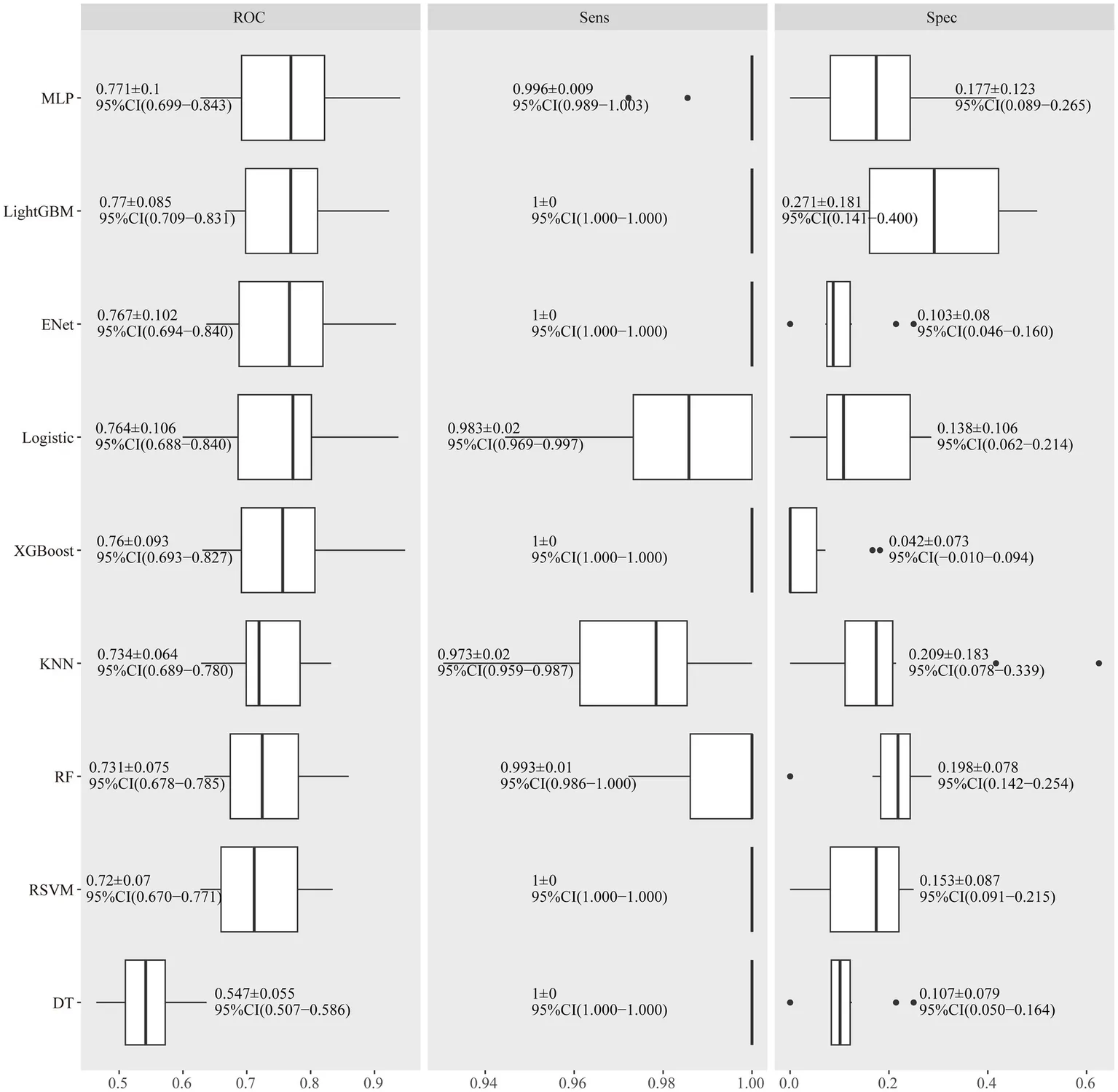
Cross-validation performance of nine machine learning (ML) models in the development set. Box-and-whisker plots showing the cross-validation performance of the nine machine learning (ML) models for predicting early neurological deterioration (END): multilayer perceptron (MLP), light gradient boosting machine (LightGBM), elastic net (ENet), logistic regression (Logistic), extreme gradient boosting (XGBoost), K-nearest neighbors (KNN), random forest (RF), reduced support vector machine (RSVM), and decision tree (DT). The three panels show the area under the receiver operating characteristic curve (AUC), sensitivity (Sens), and specificity (Spec) of each model, expressed as the mean ± SD with 95% confidence intervals, obtained by repeated cross-validation within the development set. END, early neurological deterioration; DT, decision tree; ENet, elastic net; KNN, K-nearest neighbors; LightGBM, light gradient boosting machine; MLP, multilayer perceptron; RF, random forest; AUC, area under the receiver operating characteristic curve; RSVM, reduced support vector machine; XGBoost, extreme gradient boosting.

On the development set itself, the tree-based and ensemble models attained the highest apparent AUCs—RF 0.886, KNN 0.842, and LightGBM 0.829—whereas logistic regression reached 0.787 (0.735–0.839; Figure 4A; Supplementary Table S4). This ordering reversed on the independent hold-out validation set. RF, KNN, and LightGBM each lost discrimination (AUCs 0.685, 0.665, and 0.731, respectively), while logistic regression remained the most stable model, at 0.751 (0.668–0.834; Figure 4B; Supplementary Table S4)—a fall of only 0.036 between development and validation. Logistic regression was matched only by XGBoost (0.752) and MLP (0.747), the two other models that had generalized comparatively well.

**Figure 4:**
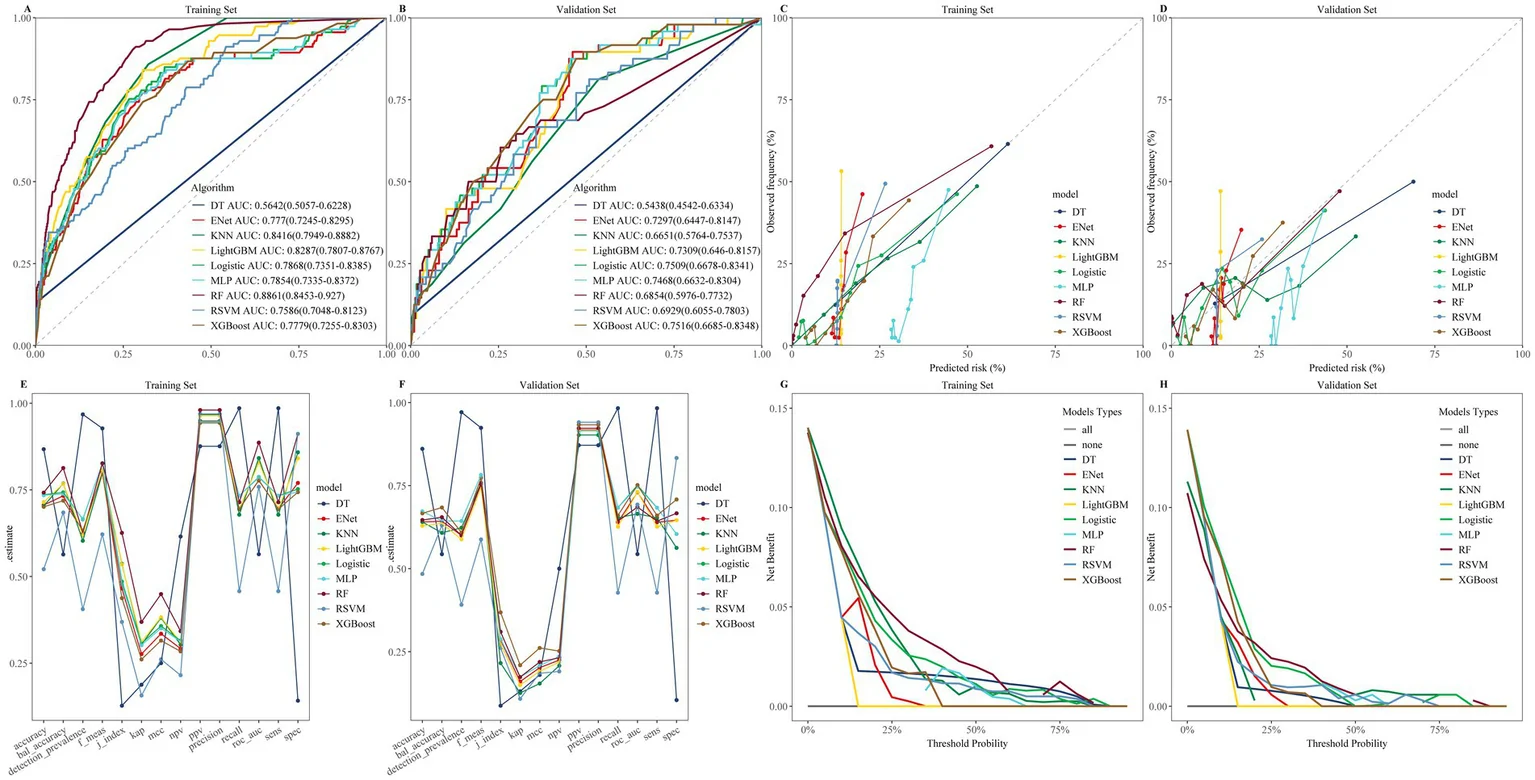
Performance of machine learning (ML) models predicting early neurological deterioration in the development and validation sets. Performance of the nine machine learning (ML) models for predicting early neurological deterioration (END) in the development (*n* = 806) and validation (*n* = 345) sets. **(A,B)** Receiver operating characteristic (ROC) curves of the nine models in the development **(A)** and validation **(B)** sets; the area under the curve (AUC) for each model is indicated. **(C,D)** Calibration plots comparing the observed and predicted risk in the development **(C)** and validation **(D)** sets. **(E,F)** Comparison of the performance metrics (accuracy, Kappa, Matthews correlation coefficient [MCC], negative and positive predictive values, precision, recall, F-measure, J index, ROC AUC, sensitivity, and specificity) across the nine models in the development **(E)** and validation **(F)** sets. **(G,H)** Decision curve analysis showing the net benefit of the models across threshold probabilities in the development **(G)** and validation **(H)** sets; the grey lines represent the treat-all and treat-none strategies. AUC, area under the curve; END, early neurological deterioration; ML, machine learning; ROC, receiver operating characteristic.

Calibration told the same story. Logistic regression had the lowest log-loss of any model on the validation set (0.347), followed by XGBoost (0.358); in contrast, the models that had overfit the development data calibrated poorly in validation, with log-loss values of 1.40 for RF and 1.20 for KNN (Supplementary Table S5), and their calibration curves departed clearly from the ideal diagonal (Figures 4C,D).

Furthermore, a comprehensive evaluation of additional performance metrics—including balanced accuracy, Kappa statistic, and F-measure—confirmed that the logistic regression model maintained consistent and robust performance across both the development and validation sets (Figures 4E,F).

DeLong’s testing on the training set revealed that the AUC of the Logistic model was significantly higher than those of prediction models constructed using other algorithms (*p* < 0.05; Supplementary Table S6). Net Reclassification Improvement (NRI) and Integrated Discrimination Improvement (IDI) analyses showed that the Logistic model performed well in reclassification and overall discriminatory ability (Supplementary Table S7).

DeLong testing on the validation set confirmed that logistic regression discriminated significantly better than DT, KNN, RF, and ENet (*p* < 0.05 for each) and was statistically indistinguishable from XGBoost, MLP, LightGBM, and RSVM (Supplementary Table S8). NRI and IDI analyses indicated that logistic regression reclassified risk at least as well as the competing high-performing models; relative to XGBoost, for instance, it gave a positive NRI of 0.154 (0.084–0.223; *p* < 0.001) and an IDI of 0.079 (0.051–0.107; *p* < 0.001; Supplementary Table S9). On decision curve analysis, logistic regression retained a favorable net benefit across the clinically relevant range of threshold probabilities in both the development and validation sets (Figures 4G,H).

On the strength of its stable discrimination, its superior calibration, its favorable net benefit, and its inherent transparency, logistic regression was chosen as the final model. At its optimal threshold, it reached a sensitivity of 0.73 and specificity of 0.75 on the development set, and 0.68 and 0.60 on the validation set (Supplementary Table S4).

### 3.6 Predictive behavior of the model

A patient-level heatmap of the final model showed how the five predictors combined to produce the predicted probability of END and how those probabilities aligned with observed outcomes (Figure 5). The model concentrated high probabilities among patients with atherosclerotic or cardioembolic stroke, anterior-circulation syndromes, older age, and atrial fibrillation, and this pattern was consistent between the development and validation sets.

**Figure 5:**
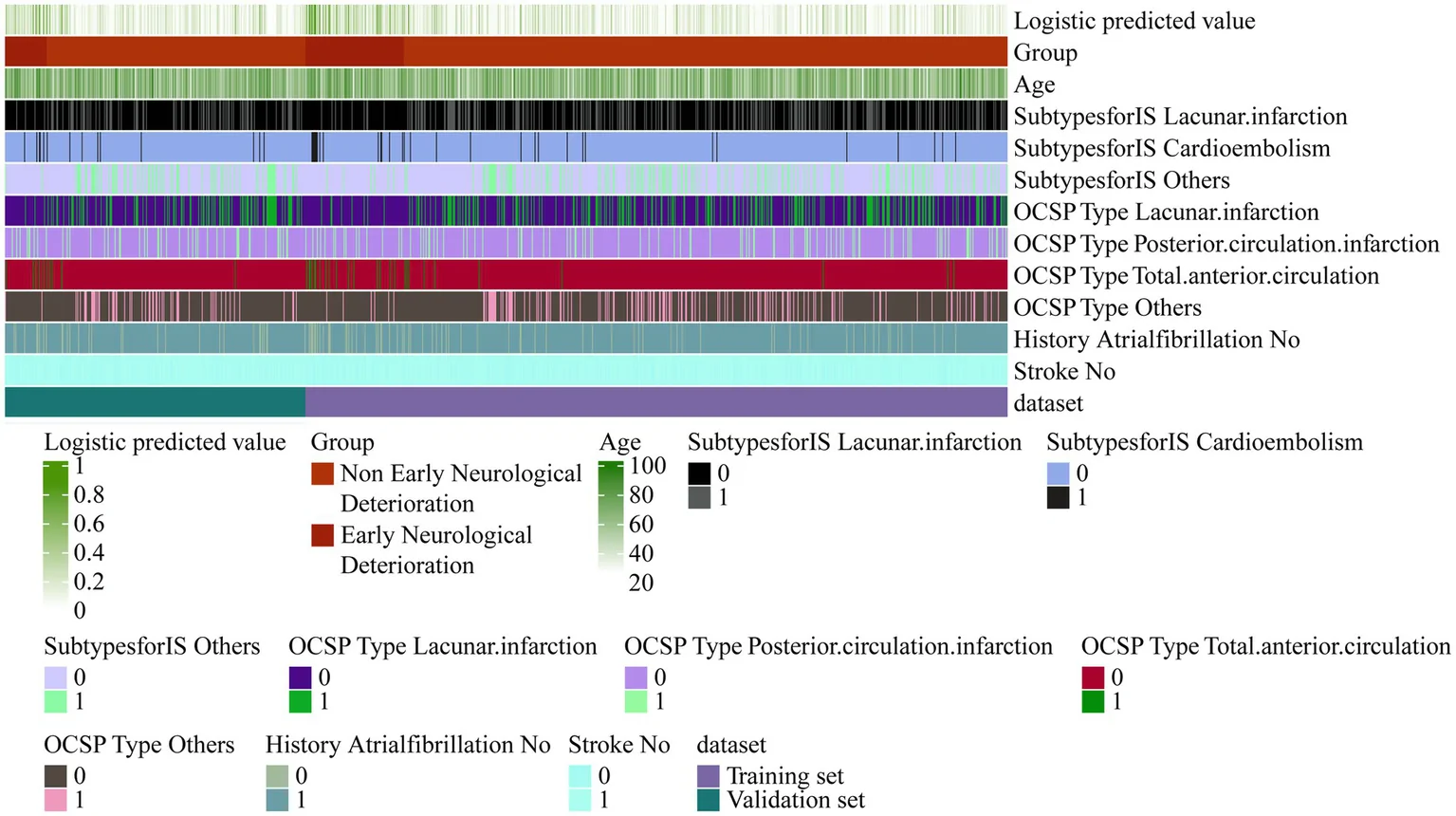
Variable heatmap of the logistic regression model for predicting early neurological deterioration. Heatmap of the predictor variables of the logistic regression (LR) model for predicting early neurological deterioration (END). Each row represents a variable and each column represents an individual patient; the color scale indicates the value of each variable. The rows comprise the predicted probability from the LR model, the true outcome group (early versus non-early neurological deterioration), age, indicator variables for ischaemic stroke subtype and OCSP classification, history of atrial fibrillation and stroke, and the dataset origin (development or validation set). The heatmap allows the variables underlying the model predictions to be visualized across the patient cohort. END, early neurological deterioration; LR, logistic regression; OCSP, Oxfordshire Community Stroke Project.

### 3.7 Model interpretation

SHAP analysis ranked the five predictors by their mean absolute contribution to the model output (Figures 6A,B). Ischemic-stroke etiological subtype was the dominant driver, followed by age, OCSP classification, previously confirmed stroke, and atrial-fibrillation history. The directions were clinically coherent: large-artery atherosclerosis and cardioembolic subtypes, total and partial anterior-circulation infarcts, and older age pushed the prediction toward END, whereas lacunar subtypes and syndromes pushed it away. Dependence plots confirmed a monotonic increase in END risk with age and clarified the effect of each categorical level (Figure 6D). At the individual level, the waterfall plot (Figure 6C) decomposed a single high-risk prediction: an atherosclerotic etiology (SHAP value +0.083) and a history of confirmed stroke (+0.066) were the largest positive contributors for that patient, with age, OCSP class, and atrial-fibrillation status adding smaller increments.

**Figure 6:**
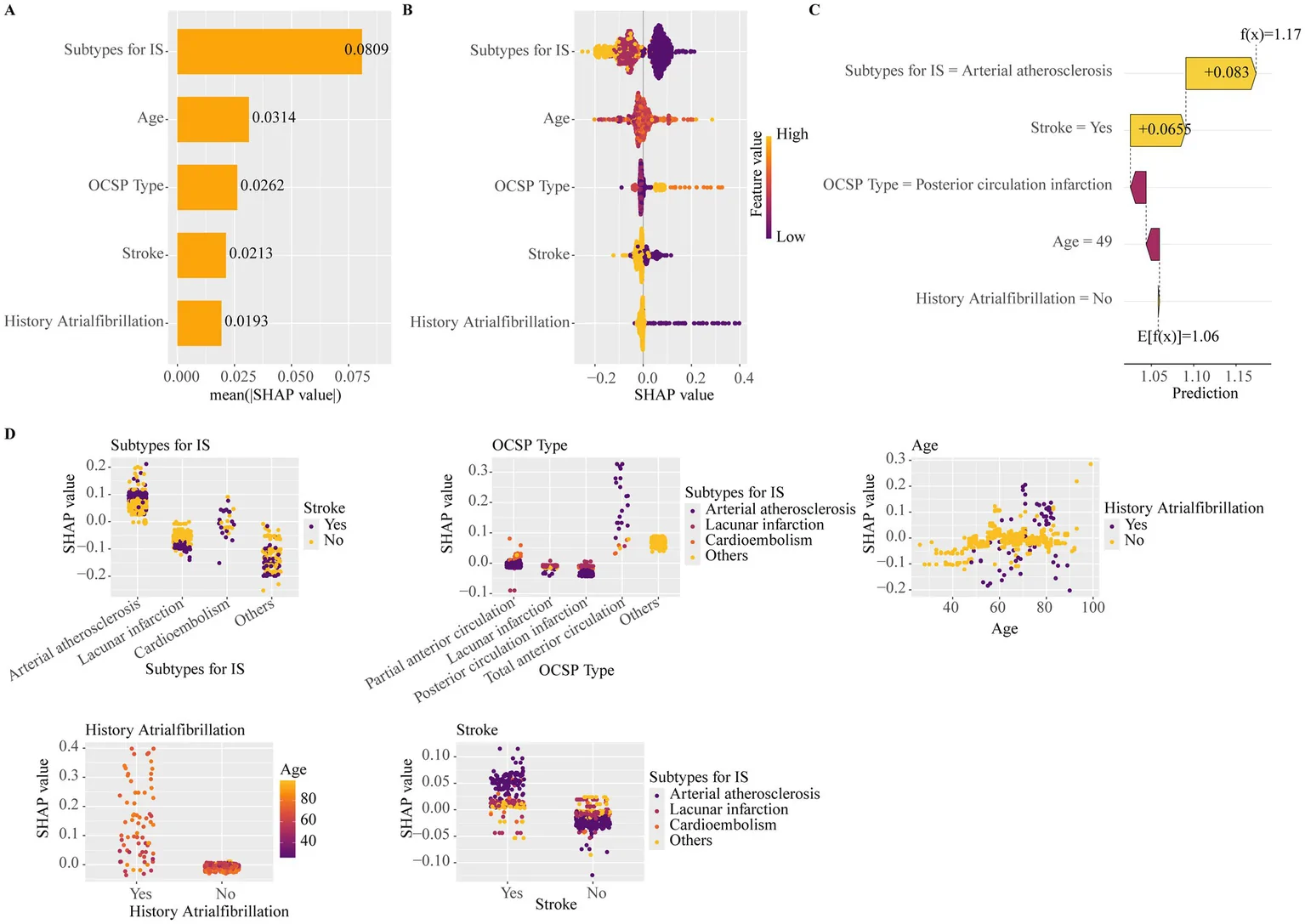
Global and local model explanation by the SHapley Additive exPlanations (SHAP) method. SHapley Additive exPlanations (SHAP) analysis of the final machine learning model for predicting early neurological deterioration (END). **(A)** Global feature importance: Predictor variables ranked by their mean absolute SHAP value (ischaemic stroke subtype, age, OCSP classification, history of stroke, and history of atrial fibrillation). **(B)** SHAP summary (beeswarm) plot in which each point represents a patient and the color indicates the feature value (yellow, high; purple, low). **(C)** Local explanation: SHAP waterfall plot for an individual patient showing how each feature contributed to the model output on the log-odds scale (*f*(*x*) = 1.17) relative to the base value (*E*[*f*(*x*)] = 1.06). **(D)** SHAP dependence plots showing the effect of individual variables on the model output. END, early neurological deterioration; OCSP, Oxfordshire Community Stroke Project; SHAP, SHapley Additive exPlanations.

### 3.8 Web calculator

The final logistic regression model was deployed as an online calculator (Figure 7). Entering the five bedside variables returns an estimated probability of END and is intended to support—not replace—clinical judgment in early risk stratification. The tool is available at https://predictingstrokeacuteexacerbation.shinyapps.io/shiny/.

**Figure 7:**
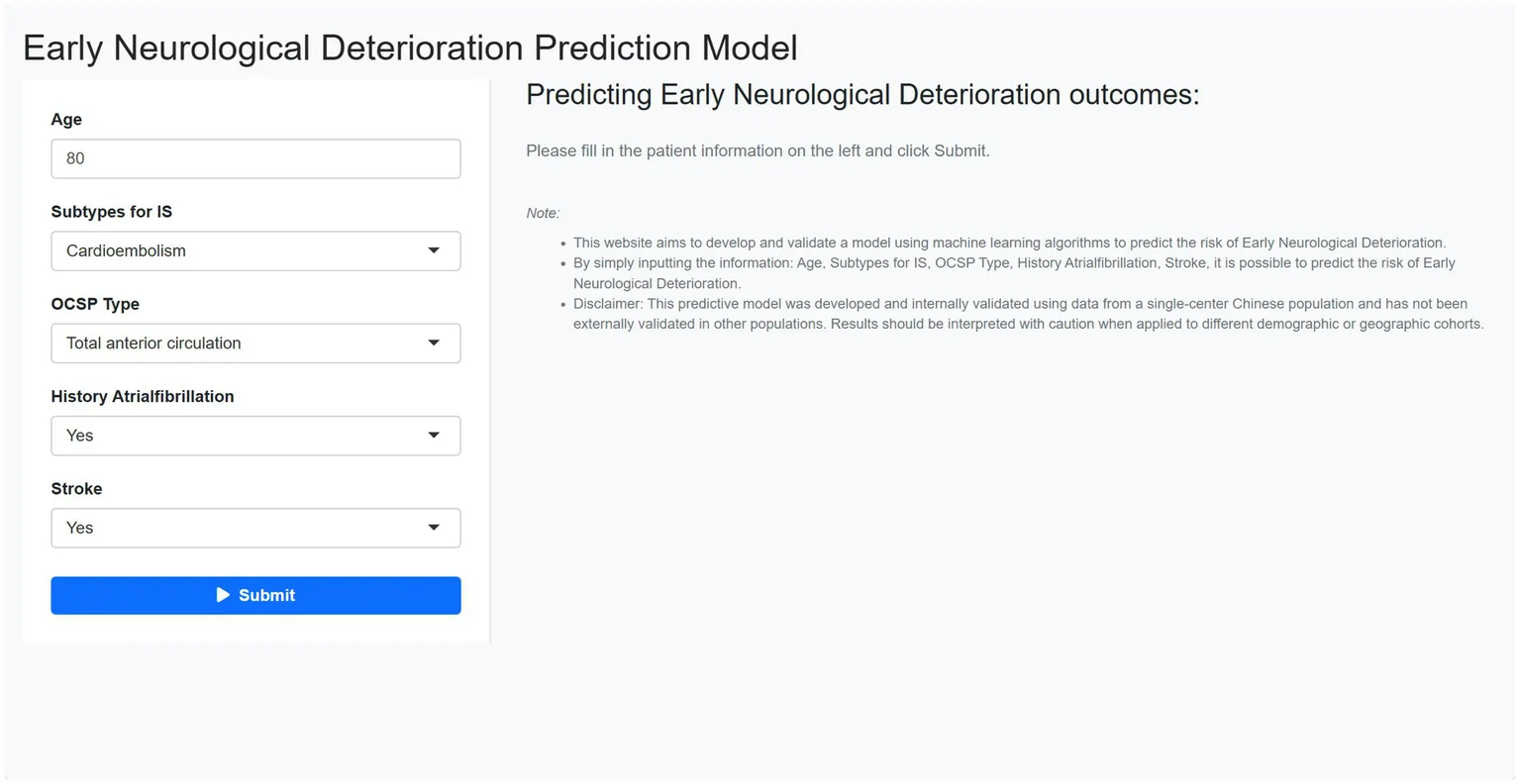
Web-based calculator for predicting early neurological deterioration. Screen capture of the web-based dynamic nomogram (risk calculator) for predicting early neurological deterioration (END). The interface allows clinicians to enter individual patient values for the model predictors and returns the corresponding predicted probability of END, supporting individualized risk assessment in clinical practice. END, early neurological deterioration.

## 4 Discussion

Based on single-center clinical data, this study developed and validated a machine learning model for predicting END after acute ischemic stroke. The final logistic regression model incorporated five routinely available clinical features (etiological subtype of ischemic stroke, OCSP classification, age, history of atrial fibrillation, and history of a confirmed stroke), demonstrated satisfactory predictive performance in the development and internal validation cohorts, with AUCs of 0.787 and 0.751, respectively. The model not only performed well on statistical metrics but also showed positive clinical net benefit in decision curve analysis. More importantly, the model’s interpretability was analyzed using the SHAP method, and an online prediction tool was constructed, laying the groundwork for its clinical translation and application.

First, the evaluation results of the model’s performance offer important insights. Compared to more complex “black-box” algorithms such as multilayer perceptron and gradient boosting machines, the logistic regression model demonstrated the best overall performance across the development and internal validation cohorts. This finding may suggest that the relationships between the selected predictors and END were adequately captured by the logistic regression model. For instance, restricted cubic spline analysis showed a significant overall positive association between age and END risk. The age-related increase in END risk may be partly attributable to poorer collateral circulation and cerebrovascular reserve, together with a greater burden of vascular comorbidities in older patients (21–23). Previous studies have shown that older age is independently associated with poorer leptomeningeal collateral status in acute ischemic stroke. Among patients with proximal middle cerebral artery occlusion, each 10-year increase in age was associated with an approximately 1.87-fold increase in the odds of inadequate collateral circulation (21). Another study involving 206 patients with intracranial large-vessel occlusion similarly found that each 10-year increase in age was associated with an approximately 1.34-fold increase in the odds of poor leptomeningeal collateral status (24). On the other hand, with a limited sample size, relatively simple models may be less prone to overfitting, which may partly explain the performance of logistic regression in the internal validation cohort. This provides a pragmatic perspective for developing clinical prediction models: more complex models are not necessarily better; the key lies in effective feature selection and appropriate model calibration (25, 26).

Second, the five core predictors identified in this study have clear clinical and pathophysiological significance and are consistent with previous research. SHAP analysis indicated that “etiological subtype of ischemic stroke” was the feature with the highest contribution. Large artery atherosclerotic and cardioembolic strokes were associated with a higher risk of END, which may be related to mechanisms such as artery-to-artery embolism and progressive hypoperfusion in the former and recurrent embolization or thrombus propagation in the latter (27, 28). Among “OCSP classifications,” total anterior circulation infarction (TACI) and partial anterior circulation infarction (PACI) had higher positive SHAP values, which may reflect more extensive anterior-circulation involvement and greater perfusion impairment (29–31). “Age” is a well-established strong predictor of poor stroke outcomes, consistent with its positive contribution to END prediction in the present study (32–34). “History of atrial fibrillation” is a key marker for cardioembolism, directly linked to the high-risk mechanism of END (35, 36). A “history of prior stroke” may indicate incompletely controlled vascular risk factors or a more extensive underlying cerebrovascular pathology in the patient. These features directly or indirectly reflect stroke severity, etiological mechanisms, and the patient’s overall vulnerability relevant to END risk.

Compared with previous studies, the main strengths of this study lie in methodological robustness and model parsimony. We systematically compared multiple machine learning algorithms and adopted an ensemble strategy (Boruta, Treebag, Bayesian method) for feature selection. This strategy enhanced the robustness of feature screening and reduced the potential bias associated with any single selection method. The final logistic regression model included only five routinely available clinical variables. Compared with some previous models that relied on complex imaging or laboratory parameters, the variables required by our model are more readily available in routine clinical practice and may facilitate future early risk assessment (37–40). Furthermore, we strictly adhered to methodological standards for prediction model research, including clear definitions of the study population and outcome, evaluation in the development and internal validation cohorts, comprehensive reporting of performance metrics (discrimination, calibration, clinical utility), and model interpretation (41). This significantly improved the reliability and transparency of the study findings.

However, this study also has several limitations. First, it is a single-center, retrospective study. Although internal validation was performed, the model’s external validity in broader populations and different healthcare settings requires further verification through multicenter, prospective studies. This logistic regression model was developed using data from a single-center Chinese cohort and has not undergone external validation in other populations. Therefore, caution should be exercised when generalizing its predictive performance to other ethnic or geographic groups. Second, the sample size was relatively limited, particularly the number of END events (161 in total). The limited number of events constrained the number of predictors and the complexity of the models that could be reliably evaluated and may have increased the risk of overfitting and instability in performance estimates. Third, although important clinical classifications were included, more granular imaging indicators (e.g., infarct core volume, perfusion mismatch ratio, collateral circulation score) and blood biomarkers were not incorporated. These variables may provide incremental predictive information beyond routinely available clinical features. Future research could integrate the clinical variables included in the present model with imaging and biomarker data and evaluate the additional predictive value of such multimodal information. Fourth, the dataset exhibited class imbalance, with END occurring in only 14.0% (161/1,151) of patients. Although the final logistic regression model showed relatively balanced sensitivity and specificity, class imbalance may still have affected the ability of some algorithms to identify END cases by favoring the majority class. Future studies using larger prospective cohorts should evaluate resampling methods such as SMOTE, cost-sensitive learning, and classification-threshold optimization to improve the identification of minority-class END cases.

The potential clinical significance of this study lies in providing an accessible tool for the early assessment of END risk after the required clinical information becomes available. The logistic regression-based model is inherently interpretable, and SHAP analysis further illustrates both overall feature contributions and individual-level predictions, thereby improving the transparency of the model outputs. The integrated online calculator is user-friendly and allows for rapid risk assessment early after patient admission. The tool may support the identification of patients who warrant closer neurological monitoring and further clinical evaluation (e.g., intensified blood pressure management, anticoagulation evaluation, consideration of endovascular therapy), thereby facilitating optimized resource allocation and personalized treatment.

## 5 Conclusion

In summary, utilizing machine learning methods, we developed and validated a risk prediction model for END based on routine clinical variables. The final logistic regression model demonstrated satisfactory predictive performance and interpretability while also being implemented as an online risk-assessment tool. This tool may support the early identification of patients at high risk of END. Prospective multicenter validation and the integration of multi-omics data may further improve the model’s predictive accuracy and support clinical risk stratification in patients with stroke.

## Data Availability

The original contributions presented in the study are included in the article/Supplementary material, further inquiries can be directed to the corresponding author.
